# Clinically relevant HIF-1α-dependent metabolic reprogramming in oropharyngeal squamous cell carcinomas includes coordinated activation of CAIX and the miR-210/ISCU signaling axis, but not MCT1 and MCT4 upregulation

**DOI:** 10.18632/oncotarget.14629

**Published:** 2017-01-13

**Authors:** Inés Sáenz-de-Santa-María, Cristóbal Bernardo-Castiñeira, Pablo Secades, Sandra Bernaldo-de-Quirós, Juan Pablo Rodrigo, Aurora Astudillo, María-Dolores Chiara

**Affiliations:** ^1^ Servicio de Otorrinolaringología, Hospital Universitario Central de Asturias, Instituto Universitario de Oncología del Principado de Asturias and CIBERONC, Universidad de Oviedo, Oviedo, Spain; ^2^ Division of Cell Biology, The Netherlands Cancer Institute, CX, Amsterdam, The Netherlands; ^3^ Servicio de Anatomía Patológica, Hospital Universitario Central de Asturias, Instituto Universitario de Oncología del Principado de Asturias, Universidad de Oviedo, Oviedo, Spain

**Keywords:** oropharyngeal squamous cell carcinoma, hypoxia inducible factor, lactate/H^+^ transporters, miR-210, ISCU

## Abstract

Metabolic reprogramming is a very heterogeneous phenomenon in cancer. It mostly consists on increased glycolysis, lactic acid formation and extracellular acidification. These events have been associated to increased activity of the hypoxia inducible factor, HIF-1α. This study aimed at defining the metabolic program activated by HIF-1α in oropharyngeal squamous cell carcinomas (SCC) and assessing its clinical impact. Global gene/miRNA expression was analyzed in SCC-derived cells exposed to hypoxia. Expression of HIF-1α, the carbonic anhydrase CAIX, and the lactate/H^+^ transporters MCT1 and MCT4 were analyzed by immunohistochemistry in 246 SCCs. Cell-based analysis revealed that HIF-1α-driven metabolic program includes over-expression of glycolytic enzymes and the microRNA miR-210 coupled to down-regulation of its target, the iron-sulfur cluster assembly protein, ISCU. pH-regulator program entailed over-expression of CAIX, but not MCT1 or MCT4. Accordingly, significant overlapping exists between over-expression of HIF-1α and CAIX, but not HIF-1α and MCT1 or MCT4, in tumor cells. Increased miR-210 and concomitant decreased ISCU RNA levels were found in ~40% of tumors and this was significantly associated with HIF-1α and CAIX, but not MCT1 or MCT4, over-expression. HIF-1α and/or CAIX over-expression was associated with high recurrence rate and low overall survival of surgically treated patients. By contrast, clinically significant correlations were not found in tumors with MCT1 or MCT4 over-expression. This is the first study that provides *in vivo* evidences of coordinated activation of HIF-1α, CAIX, miR-210 and ISCU in carcinoma and association with poor prognosis, a finding with important implications for the development of metabolic-targeting therapies against hypoxia.

## INTRODUCTION

Malignant cell transformation requires metabolic reprogramming to support anabolic cell growth. To fulfill their biosynthetic demands, cancer cells increase the uptake of glucose and reprogram carbon metabolism by the decoupling of glycolysis from the oxidative phosphorylation [1]. The glucose-derived pyruvate generated in cancer cells, rather than being oxidized in mitochondria, is transformed into lactate in the cytosol and is subsequently exported to the micro-environment. This metabolic shift with production of lactate even in the presence of sufficient oxygen is known as the Warburg effect [2] which is a highly regulated metabolic state that diverts glycolytic intermediates into branching pathways that allow the generation of biosynthetic precursors [3].

The secretion of lactate to the extracellular space is tightly regulated by the overexpression of several pumps and ion channels including monocarboxylate transporters (MCTs). MCTs are specialized for the co-transport of monocarboxylates and H^+^, which causes acidification of the tissue microenvironment. The MCT family includes 14 different members of which MCT1 [4, 5] and MCT4 [6] are specialized for the co-transport of lactate and H^+^. MCT1 is implicated in both lactate export and import and has been implicated in the transfer of metabolites between tumour cells and stromal cells [7]. MCT4, which has a lower affinity for lactate than MCT1, almost exclusively acts as a lactate-exporting protein, thus favoring the glycolytic flux in cancer cells [8, 9]. Besides lactate, oncogenic metabolism also generates an excess of CO_2_ that contributes to the extracellular acidification. CO_2_ diffuses into the extracellular space, where it is converted to H^+^ and HCO_3_^-^ by extracellular carbonic anhydrases (CAs) [10]. HCO_3_^−^ anions can then be recaptured by the tumour cells through HCO_3_^−^ transporters to assist with intracellular-pH buffering [11, 12]. These proteins, coupled with other modifiers of the intracellular pH, actively contribute to the development of tumor cells that have a more alkaline intracellular pH than normal cells, despite being surrounded by an acidic stromal microenvironment. The generation of this unique pH gradient likely represents a key factor in promoting the capacity of the tumor to invade and metastasize [13].

Many metabolic pathways involved in the reprogramming of cancer cells and pH regulation are closely linked to the metabolic changes associated with hypoxia. In this regard, the hypoxia inducible factor-1α (HIF-1α) which, together with HIF-2α, transcriptionally activates target genes in response to hypoxic conditions, functions as an essential driver of metabolic reprogramming [14]. HIF-1α induces genes involved in glucose uptake, glycolytic pathway, and acid-base regulation including MCT4 [6] and CAIX [15]. HIF-1α also restricts mitochondrial respiration by repression of the iron-sulfur cluster assembly protein (ISCU) which is a target of the HIF-1α-induced microRNA, miR-210 [16–18]. ISCU facilitates the assembly of iron-sulfur clusters, the prosthetic groups that are critical for mitochondrial oxidation-reduction reactions [19]. Thus, the miR-210-mediated disruption of ISCU promotes a shift from mitochondrial oxidative phosphorylation to increased rates of glycolysis coupled with lactic acid formation and intracellular acidification [20].

Although has now been recognized that reprogramming of cellular metabolism is essential for tumorigenesis [3], the cancer-induced changes in the expression of metabolic genes are very heterogeneous across different tumor types [21] and, thus, focused analysis of essential metabolic transformations in each specific cancer type is required to fully understand the link between cancer and metabolism. Specifically, activation of the HIF-1α/miR-210/ISCU signaling pathway has not been demonstrated in most cancer types and whether cancer cells coordinately activate hypoxia/HIF-related pH-regulating proteins and the miR-210/ISCU signaling axis is presently unknown. This study aimed to define the gene and microRNA metabolic programs activated by hypoxia/HIF-1α in squamous cell carcinomas of the oropharynx. In addition, we assessed whether, in these primary tumors, the HIF-1α/miR-210/ISCU signaling pathway is activated and whether this axis, the lactate transporters and the CAIX pH-regulator are coordinately regulated. We further assessed their clinical impact in surgically treated squamous cell carcinomas of the oropharynx.

## RESULTS

### Global changes in metabolic gene expression and microRNA levels induced by HIF-1α

We first sought to identify the specific metabolic programs activated by hypoxia in SCCs. To this end, microarray gene profiling was performed in head and neck derived-SCC38 cells treated with control-siRNA or HIF-1α-siRNA and exposed to normoxia (21% O_2_) or hypoxia (1% O_2_) for 16 hours. Previous experimental data had revealed time-dependent response to hypoxia of SCC cells with significant increase of all tested HIF-1α targets at 16 hours of hypoxia treatment (Supplementary Figure 1A). The inhibition of hypoxic induction of HIF-1α -target genes, caused by HIF-1α-siRNA treatment, was also verified to be operative in our experimental conditions (Supplementary Figure 1B).

**Figure 1:**
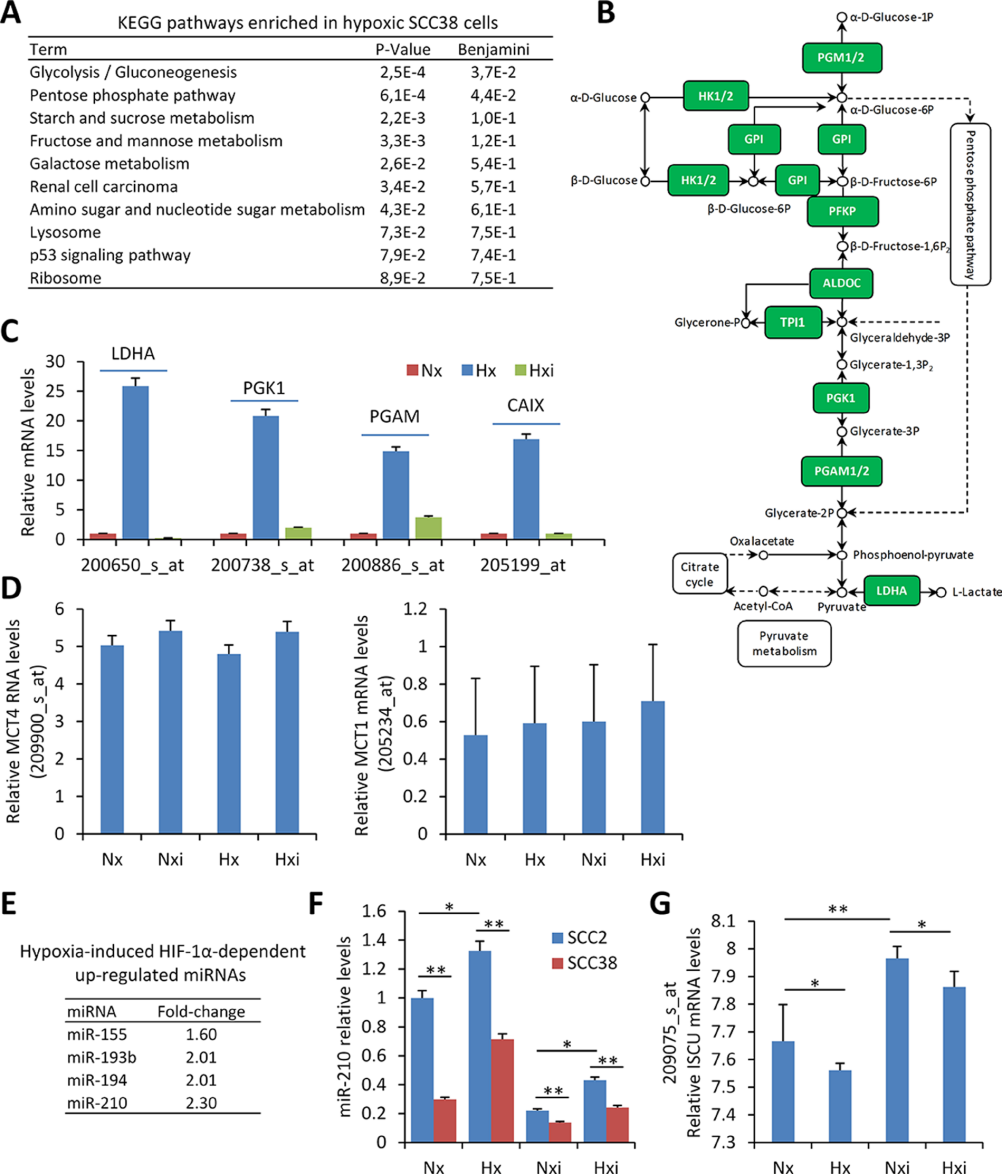
microRNAs and transcriptional-metabolic programs activated by hypoxia in SCC-derived cells (**A**–**D**) Four replicates of SCC38 cells treated with control- or HIF-1α siRNAs for 24 h were exposed to normoxic (Nx: control siRNA, Nxi: HIF-1α siRNAs, 21% O_2_) or hypoxic (Hx: control siRNA, Hxi: HIF-1α siRNAs, 1% O_2_) environments for 16 h prior to total RNA isolation and microarray analysis. (A, B) KEGG pathway enrichment in hypoxic versus normoxic SCC38 cells. The diagram of the glycolysis KEGG pathway is provided in B highlighting (green) the up-regulated HIF-1α-related genes identified in SCC38 cells exposed to hypoxia in comparison with normoxia. (C, D) Expression values of lactate dehydrogenase A (LDHA), phosphoglycerate kinase 1 (PGK1), phosphoglycerate mutase 1 (PGAM), CAIX, MCT4, and MCT1 detected by microarray analysis with 200650_s_at, 200738_s_at, 200886_s_at and 205199_at, 209900_s_at, 205234_at probesets, respectively, in cells incubated under the indicated conditions. (**E**, **F**) Four replicates of SCC38 and SCC2 cells treated with control- or HIF-1α siRNAs for 24 h were exposed to normoxic (Nx: control siRNA, Nxi: HIF-1α siRNAs, 21% O_2_) or hypoxic (Hx: control siRNA, Hxi: HIF-1α siRNAs, 1% O_2_) environments for 16 h prior to total RNA isolation and miRNA analysis. (E) List of hypoxia-induced HIF-1α-dependent up-regulated miRNAs. (F) miR-210 relative levels detected by RT-qPCR in the indicated cells. (**G**) Expression values of ISCU mRNA detected in SCC38 cells by microarray analysis with 209075_s_at probeset. *p < 0.05, **p < 0.005.

The microarray results from four replicates for each condition were pooled together for the analysis. As shown in Figure 1A and 1B, the Kyoto Encyclopedia of Genes and Genomes (KEGG) pathway involved in glycolysis/gluconeogenesis was found as the most significantly enriched in hypoxic versus normoxic cells. This includes TPI, HK, LDHA, PGK, PGAM genes which are known HIF-1α target genes. As expected, these genes were not found up-regulated by hypoxia when SCC38 cells were treated with HIF-1α-directed siRNAs (Figure 1C). In addition to that, the pH-regulating enzyme, CAIX, was found among the genes most highly up-regulated by hypoxia in a HIF-1α-dependent manner. However, other pH-regulating genes, such as MCT1 and the reported target of HIF-1α, MCT4, were not significantly up-regulated by hypoxia.

To identify microRNAs regulated by hypoxia in a HIF-1α-dependent manner, miRNA profiling was performed in two SCC-derived cell lines (SCC2 and SCC38) exposed to hypoxia or normoxia after treatment with either control- or HIF-1α-siRNAs. SCC2 cells are known to harbor HIF-1α gene amplification and constitutive normoxic HIF-1α protein accumulation which is not further increased by hypoxic treatment [22]. Accordingly, in comparison with SCC38 cells, SCC2 cells overexpress HIF-1α target genes in normoxia which is abrogated by HIF-1α-siRNA-mediated reduction of HIF-1α expression [22]. Thus, SCC2 cells served as a positive control to study the role of HIF-1α on hypoxic regulation of miRNAs in SCCs. Four miRNAs fulfilled the criteria for HIF-1α targets at a cut-off of ≥ 1.5- fold change (Figure 1E, 1F). Of these, miR-155 and miR-210 have already been reported to be regulated by HIF-1α under hypoxic conditions [23, 24] and miR-193 has also been associated with hypoxia [25] although an association with HIF-1α has not been so far reported. In SCC-derived cells, we previously demonstrated that one of miR-210 targets, ISCU, is inversely correlated with miR-210 expression and is likely involved in the downregulation of mitochondria complex II activity [26]. Analysis of ISCU levels in the microarray data revealed a significant reduction by hypoxia and over-expression by HIF-1α siRNAs in both normoxic and hypoxic conditions (Figure 1G). The inverse correlation of miR-210 and ISCU expression was validated in an independent cell line (Supplementary Figure 2).

Overall, the data confirm that hypoxia induces a HIF-1α-dependent gene and microRNA expression signature consistent with the Warburg effect in SCC-derived cells. This is accompanied by over-expression of the CAIX enzyme that contributes to acidification of the extracellular microenvironment while maintaining neutral intracellular pH. However, other pH-regulating enzymes such as MCT1 and MCT4 were not significantly up-regulated by hypoxia in this cell-based system. To more clearly delineate the roles and the interconnections between HIF-1α-related metabolic changes and pH-regulating enzymes in tumor behavior, we analyzed the expression of HIF-1α, CAIX, MCT1 and MCT4 proteins and their associations with each other and with a miR-210/ICU signaling pathway in patient-derived primary SCCs.

### HIF-1α, CAIX, MCT1 and MCT4 expression in oropharyngeal SCCs

**Figure 2:**
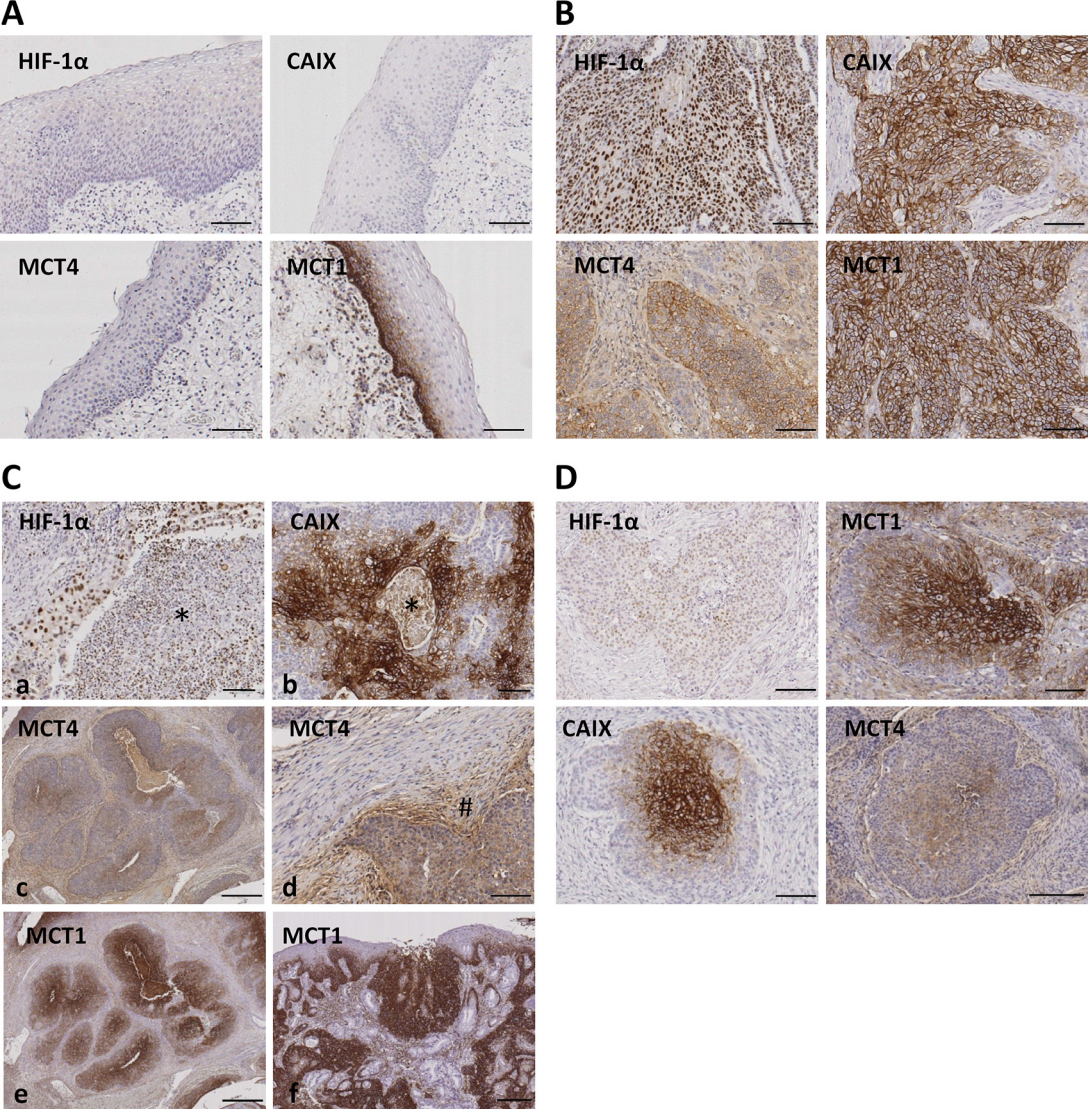
Immunohistochemical staining for HIF-1α, CAIX, MCT4 and MCT1 in normal oropharyngeal normal mucosa and oropharyngeal SCCs **(A)** Representative images of normal mucosa adjacent to SCC tissues showing absence of immunostaining for HIF-1α, CAIX and MCT4. The basal layer of the normal epithelia is specifically immunolabeled with anti-MCT1 antibody. **(B)** Representative images of oropharyngeal SCCs showing immunolabeling of cancer cell nuclei (HIF-1α) or cancer cell membranes (MCT4, CAIX, MCT1). **(C)** Representative images of oropharyngeal SCCs showing immunolabeling of cancer associated stromal cells located in perinecrotic areas indicated by asterisks (HIF-1α and CAIX; a and b) or located around tumor niches indicated by pound sign (#) (MCT4; c-d). Note that stromal cells located far from the tumor nests are not immunolabeled for MCT4 (d). MCT1 immunolabeling of cancer, but not stromal, tissue (f) and dysplastic epithelia (g). **(D)** Serial tumor sections showing Immunostainings for HIF-1α, CAIX, MCT4 and MCT1 in the central area of tumor nests. Original magnifications, x100 (A, B, D, Ca, Cb, Cd), x500 (Cc, Ce, Cf).

A total of 246 SCC samples from the oropharynx were included in this study. Clinical features are described in Table 1. As shown in Figure 2A, HIF-1α, CAIX and MCT4 immunostainings were not detected in normal mucosa, whereas MCT1 immunostaining was strongly detected in the basal layer of the normal epithelia, in agreement with previous reported data [27]. In tumor cells, as expected, HIF-1α immunostaining was confined to the nuclei whereas CAIX, MCT1 and MCT4 decorated the tumor cell membranes (Figure 2B). No cytoplasmic staining was observed with any of the antibodies.

**Table 1:** Clinico-pathological features of the patients included in this study

	*n*	percentage
**Age (years)**		
> 58	134	53.8
< 58	115	46.2
**Sex**		
male	240	96.4
female	9	3.6
**Location**		
base of tongue	108	43.4
tonsil	141	56.6
**pT classification**		
T1 + T2	80	32.1
T3 + T4	169	67.9
**pN clasiffication**		
N0	62	25
≥ N1	186	75
**Histopathologic grade**		
well differentiated	114	46
poorly differentiated	134	54
**Distant metastasis**		
No	197	79.1
Yes	52	20.9
**Disease stage**		
I + II	28	11.2
III + IV	221	88.8
**Recurrence**		
No	100	40.2
Yes	149	59.8
**HPV status**		
negative	240	96.8
positive	8	3.2
**Treatment modalily**		
surgery	82	32.9
Surgery + postoperative radiotherapy	167	67.1

HIF-1α and CAIX staining was detected in stromal cells exclusively when these were located inside and next to areas of necrosis (Figure 2C). MCT4 staining, in contrast, was found in stromal cells surrounding the tumor niches but not in those located far from the tumor area thus indicating a specific staining of cancer associated fibroblasts and inflammatory cells (Figure 2C). Staining of MCT1 was exclusively found in cancer cells and in the hyperplasic and dysplastic epithelia adjacent to the tumors thus indicating that MCT1 over-expression is an early event in tumorigenesis (Figure 2C). In this report, only cancer cell immunostaining was considered for quantification and correlation analysis.

**Figure 3:**
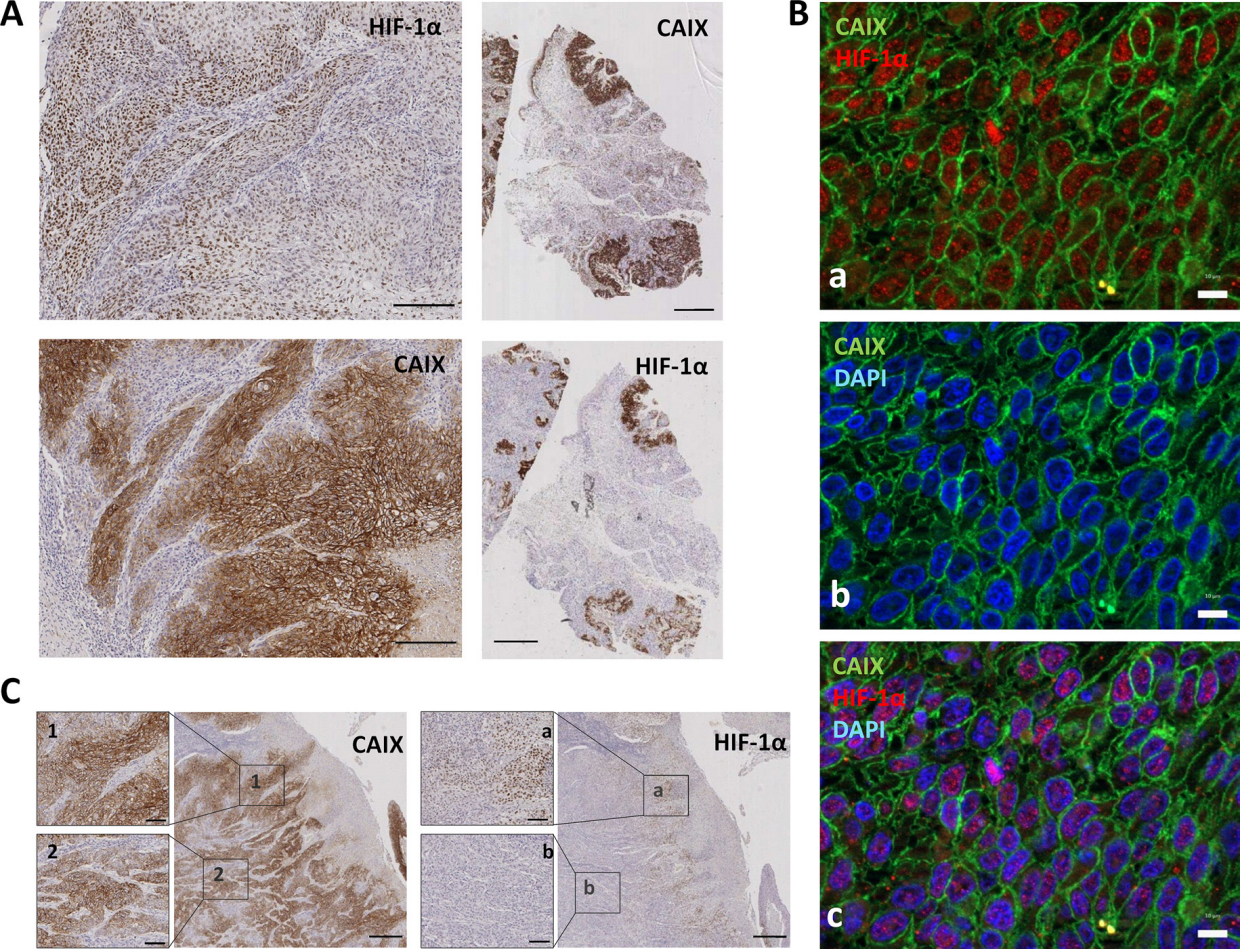
HIF-1α is consistently co-expressed with CAIX in tumor cells **(A)** Representative photomicrographs of serial tumor sections of a SCC tumor biopsy immunostained for HIF-1α and CAIX. Original magnifications ×200 (left pictures), ×2000 (right pictures). **(B)** Double immunofluorescence labeling of SCC tissues for HIF-1α (red) and CAIX (green); cell nuclei were stained with 4′,6-diamidino-2-phenylindole (DAPI) (blue). Images show that nuclear-HIF-1α and membrane-CAIX immunostainings co-localize in the same cancer cells. Scale bars, 20 μm **(C)** Representative immunostaining of serial tumor sections of a SCC tumor biopsy showing a more extensive staining for CAIX than for HIF-1α. 1, 2, a, and b are higher magnifications of the areas outlined in the adjacent panels. Original magnifications ×500 and ×100.

**Figure 4:**
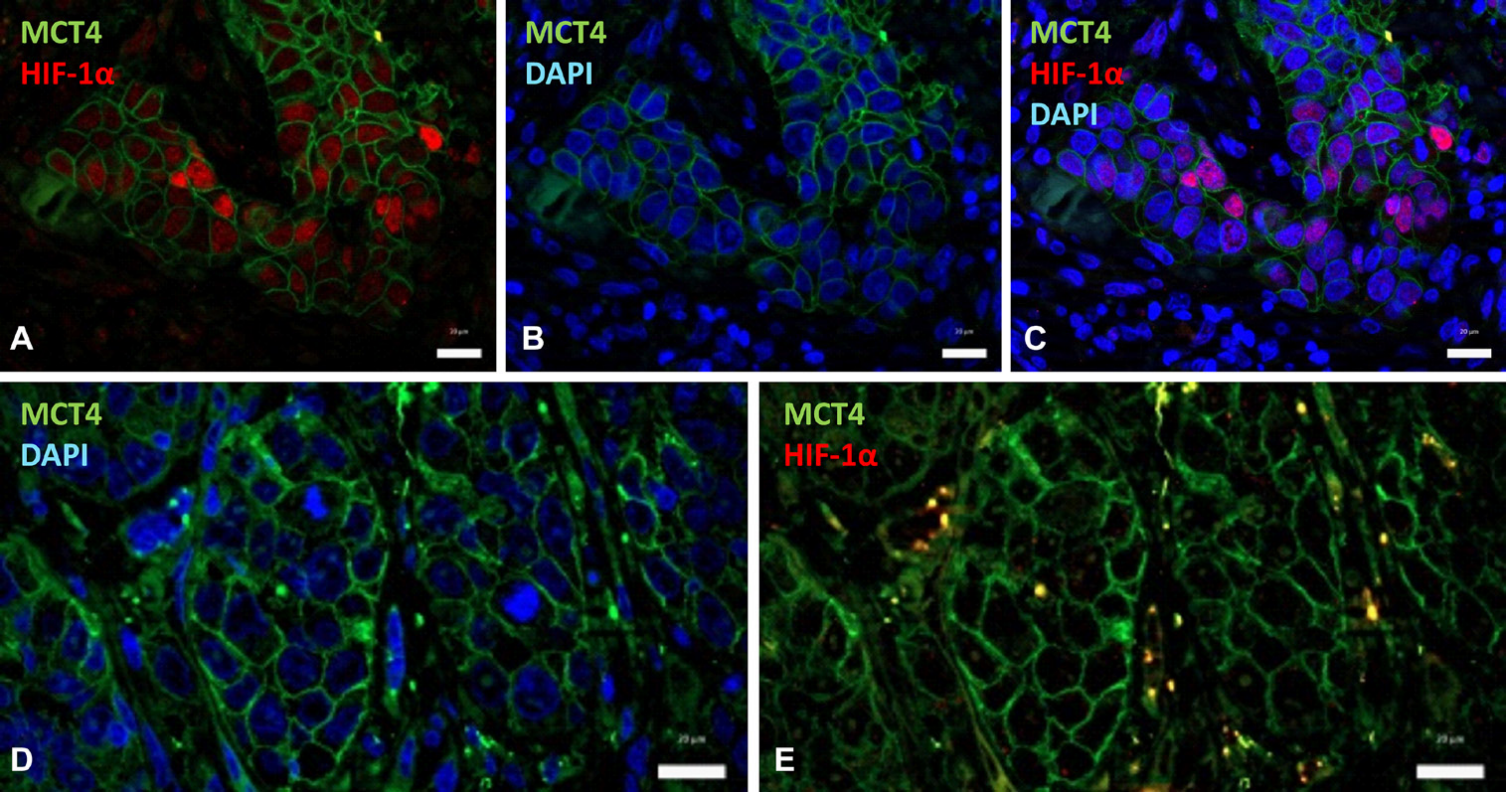
HIF-1α partially overlaps with MCT4 over-expression Double immunofluorescence labeling of SCC tissues for HIF-1α (red) and MCT4 (green); cell nuclei were stained with 4′,6-diamidino-2-phenylindole (DAPI) (blue). **(A–C)** Images show that nuclear-HIF-1α and membrane-MCT4 may co-localize in the same cancer cells. **(D, E)** Representative images of a different tumor region that contains negative nuclear-HIF-1α and positive membrane-MCT4 immunostainings. Scale bars, 20 μm.

Regarding geographic distribution of immunostained cancer cells within the tumor sections, positivity for HIF-1α, as well as for CAIX, MCT4 and MCT1 were detected in perinecrotic areas and in the center of tumor nests likely in relation with hypoxia (Figure 2D). Apart from those areas, a more extensive labeling of the tissues was found with the four antibodies. With regard to HIF-1α and CAIX, in agreement with previous reports [15], an almost complete overlap of the two hypoxic biomarkers was detected, as shown by immunostaing of serial tumor slices (Figure 3A). Co-inmunofluorescence analysis also confirmed co-expression of HIF-1α and CAIX in the same tumor cells (Figure 3B). Nevertheless, CAIX was occasionally found in HIF-1α-negative areas, possibly because reoxygenation leads to degradation of HIF-1α, but not of the highly stable CAIX protein (Figure 3C). In contrast to HIF-1α and CAIX, an incomplete overlapping of HIF-1α with MCT4 or MCT1 was frequently observed. Co-inmunofluorescence analysis of HIF-1α and MCT4 showed that although, in some tumor areas, both proteins were co-expressed in the same tumor cells, this overlapping was imperfect; tumor areas containing MCT4 positive cells and complete absence of HIF-1α positive nuclei in and around MCT4 positive cells were frequently detected (Figure 4). Regarding MCT1, positively stained cells were distributed homogeneously in the tumor sections (Figure 2C) so that were detected close to, but not preferentially associated to, areas of necrosis or the centers of tumor nests. Thus, our data indicate that hypoxia and MCT4/MCT1-induced lactic acidosis are partially linked, i.e. the two phenomena may occur at the same and also in different tumors sites.

Because the staining with the HIF-1α, CAIX, MCT1 and MCT4 antibodies showed variability in the spatial distribution and the intensity, samples were scored as fractions of positive cells as well as according to intensity of positive cells. The median value was selected as the cutoff to define highly immunostained samples (above the cutoff) and weakly immunostained samples (below the cutoff). Results were obtainable in 245 patients (98.4%) for HIF-1α, 246 patients (98.8%) for CAIX, 75 patients (30%) for MCT4 and 77 patients (31%) for MCT1. High staining of HIF-1α, CAIX, MCT4 and MCT1 were detected in 24.5% (60/245), 50% (123/123), 48% (36/75), and 45% (35/77) of samples, respectively. Using the expression score as a continuous variable, there was a high degree of correlation between HIF-1α expression and CAIX expression (correlation coefficient *r* = 0.363, *p* < 0.0001) and between HIF-1α and MCT1 (correlation coefficient *r* = 0.231, *p* < 0.044). MCT4 and MCT1 were also more frequently overexpressed in tumors with high levels of CAIX (MCT4: correlation coefficient *r* = 0.281, *p* = 0.015; MCT1: correlation coefficient 0.271, p = 0.018). No significant correlations were found between HIF-1*α* and MCT4 (correlation coefficient *r* = 0.167, *p* = 0.155) or between MCT1 and MCT4 (correlation coefficient *r* = 0.137, *p* = 0.241).

### Evidences for activation of the miR-210/ISCU signaling axis in hypoxic oropharyngeal SCCs

Analysis of miR-210 and ISCU mRNA could be performed in 14 tumors for which high quality RNA was available (Table 2). Three samples from normal mucosa obtained from non-cancerous patients were used as control. As compared with miR-210 levels in normal mucosa, 4 out of 14 samples (40%) showed miR-210 overexpression. Low ISCU mRNA levels were found in 8/14 (57.1%) of samples. None of the samples had low miR-210 levels or ISCU over-expression as compared with control samples. miR-210 and ISCU RNA levels were inversely correlated (*p* = 0.040). Comparison of miR-210 with HIF-1α, CAIX, MCT1 and MCT4 protein levels revealed a positive association between miR-210 over-expression and HIF-1α (*p* = 0.015) and CAIX (*p* = 0.052) but not MCT4 (*p* = 0.853) or MCT1 (*p* = 0.725) over-expression. ISCU down-regulation correlated inversely with CAIX (*p* = 0.016) and HIF-1α (*p* = 0.040) over-expression. There was no correlation between ISCU and MCT1 (*p* = 0.279) or MCT4 (*p* = 0.725).

**Table 2:** Correlation between miR-210, ISCU and HIF-1α, CAIX, MCT4 and MCT1

	miR-210				ISCU		
	< 1.6	≥ 1.6	*P*		≥ 0.75	< 0.75	*P*
HIF-1α				HIF-1α			
Low	9 (64%)	1 (7%)	0.015	Low	6 (44%)	4 (29%)	0.04
High	1 (7%)	3 (22%)		High	0 (0%)	4 (29%)	
CAIX				CAIX			
Low	8 (57%)	1 (7%)	0.052	Low	6 (44%)	3 (21%)	0.016
High	2 (14%)	3 (22%)		High	0	5 (35%)	
MCT4				MCT4			
Low	5 (39%)	2 (15%)	0.853	Low	3 (23%)	4 (31%)	0.725
High	4 (31%)	2 (15%)		High	2 (15%)	4 (31%)	
MCT1				MCT1			
Low	7 (54%)	1 (8%)	0.071	Low	4 (31%)	4 (31%)	0.279
High	2 (15%)	3 (23%)		High	1 (7%)	4 (31%)	
ISCU							
≥ 0.75	6 (44%)	0 (0%)	0.04				
< 0.75	4 (29%)	4 (29%)					

We then extended the RNA analysis to a total of 35 oropharyngeal SCC samples for which CAIX expression data were available. In this series, the inverse correlation between miR-210 and ISCU mRNA levels was maintained (Pearson R −0.4855, R square 0.2357, 95% confidence interval −0.7531 to −0.08028, p value two tailed 0.0220): 75% (6/8) of samples with miR-210 over-expression had low levels of ISCU mRNA whereas 35% (7/20) of samples had low ISCU but not increased miR-210 levels. In addition, samples with high miR-210 levels or low ISCU mRNA levels had significantly (*p* < 0.001 and *p* = 0.019, respectively) higher CAIX immune-scores (CAIX levels: 144 ± 89.7 for high miR-210-expressers and 98.6 ± 92.6 for low ISCU-expressers) than samples lacking miR-210 over-expression or ISCU down-regulation (CAIX levels: 37.7 ± 43.9 for miR-210 and 40.4 ± 46.6 for ISCU). No correlations between MCT1 or MCT4 and miR-210 or ISCU were observed. Thus, the data in this validation set was comparable to that of the previous series.

**Figure 5:**
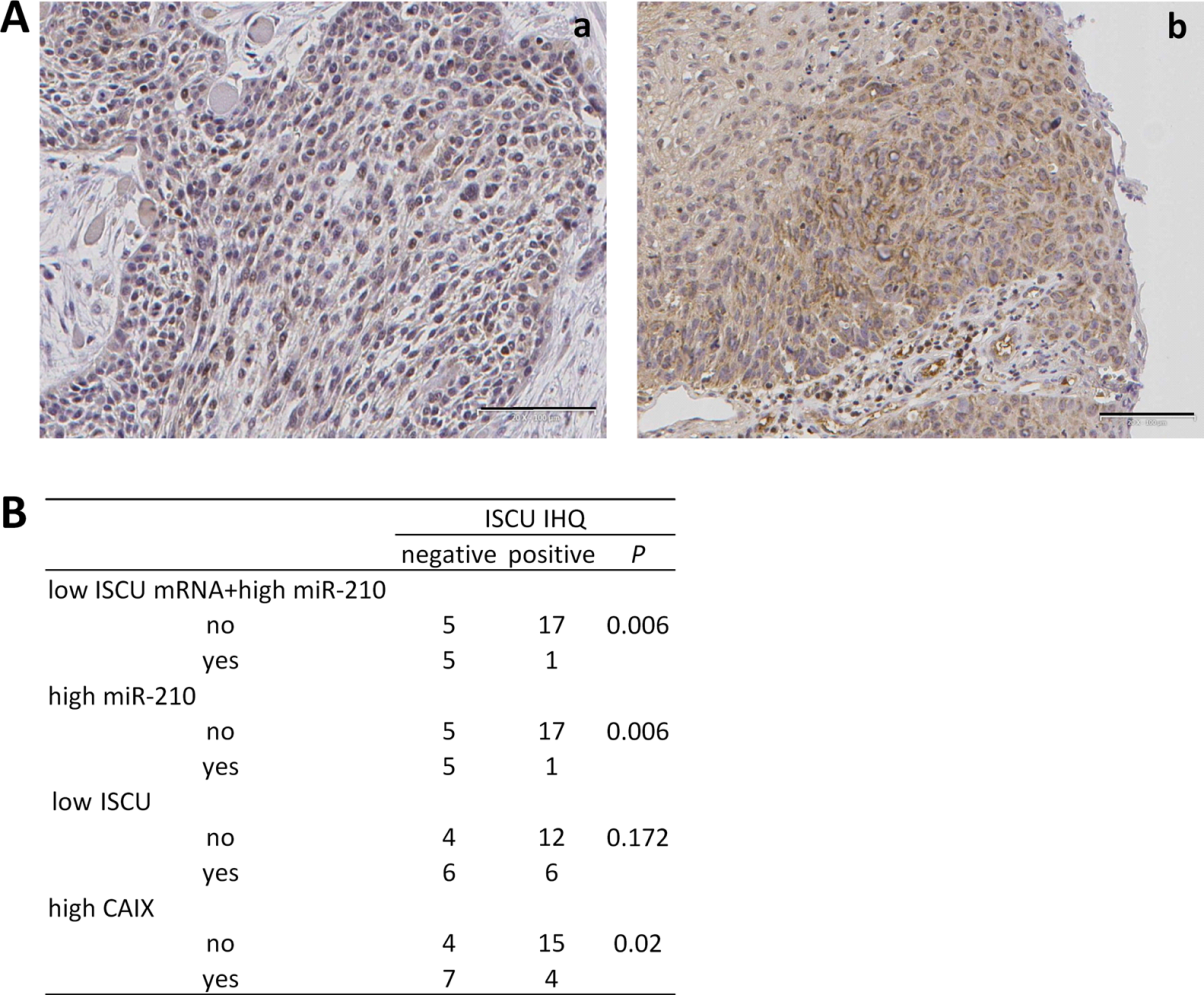
Correlation between decreased ISCU protein and increased miR-210 levels **(A)** Representative photomicrographs of SCC tumor biopsies immunostained for ISCU. Images correspond to tumor tissues with (a) and without (b) low ISCU mRNA+high miR-210 levels. Original magnifications ×100. **(B)** Correlation between ISCU mRNA and protein levels, miR-210 and CAIX.

We additionally analyzed ISCU protein expression in 28 of the tumor samples with available miR-210 and ISCU data. This analysis revealed that absence of ISCU immunostaining was significantly more frequent in samples with high miR-210 plus low ISCU mRNA expression (*p* = 0.006) than in those lacking these gene alterations (Figure 5). It also significantly correlated with high miR-210 expression (*p* = 0.006). Although the percentage of samples showing absence of ISCU protein was higher in samples with low mRNA ISCU levels than in samples without decreased mRNA ISCU levels, the differences were not statistically significant (*p* = 0.172). On the other hand, absence of ISCU protein significantly correlated with high CAIX expression levels (*p* = 0.020), an association that increased when higher CAIX immunoscores were used as cutoff values (CAIX immunoscore = 40, *p* = 0.007; CAIX immunoscore = 80, *p* = 0.002).

### Protein/miR-210 expression and clinicopathological variables

Comparison of the immunohistochemical data and clinicopathological variables revealed that the only statistically significant association was between tumor size and CAIX or HIF-1α overexpression (Table 3). No significant correlations were found between MCT1 or MCT4 over-expression and clinicopathological variables. Although in a limited number of patients, which hampers reaching meaningful clinical significance of miR-210 over-expression, our data also revealed that miR-210 over-expression correlated with tumor size (*p* = 0.040) and with distant metastasis (*p* = 0.052) (Table 3).

**Table 3:** Correlations of HIF-1α, CAIX, MCT1, MCT4 and miR-210 expression and patients and tumor variables

	HIF-1α				CAIX				MCT1				MCT4				miR-210			
	*n*	low	high	*P* value	*n*	low	high	*P* value	*n*	low	high	*P* value	*n*	low	high	*P* value	*n*	< 1.6	≥ 1.6	*P* value
**pT classification**																				
T1+T2	79	66 (27%)	13 (5%)	0.044	123	48 (19%)	75 (31%)	0.020	21	13 (17%)	8 (10%)	0.427	20	13 (17%)	7 (9%)	0.174	6	6 (42%)	0 (0%)	0.040
T3+T4	166	119 (49%)	47 (19%)		123	31 (13%)	92 (37%)		56	29 (38%)	27 (35%)		55	26 (35%)	29 (39%)		8	4 (29%)	4 (29%)	
**pN clasiffication**																				
N0	62	45 (18%)	140 (18%)	0.535	62	34 (14%)	28 (11%)	0.378	18	11 (14%)	7 (9%)	0.523	17	10 (13%)	7 (9%)	0.522	3	3 (21%)	0 (0%)	0.217
≥N1	183	17 (7%)	43 (17%)		184	89 (36%)	95 (39%)		59	31 (41%)	28 (36%)		58	29 (39%)	29 (39%)		11	7 (50%)	4 (29%)	
**Histopathologic grade**																				
well differentiated	111	85 (35%)	26 (11%)	0.801	112	57 (23%)	55 (22%)	0.753	32	18 (23%)	14 (18%)	0.800	31	17 (23%)	14 (19%)	0.680	5	5 (35%)	0 (0%)	0.078
poorly differentiated	133	100 (41%)	33 (13%)		133	65 (27%)	68 (28%)		45	24 (32%)	21 (27%)		44	22 (29%)	22 (29%)		9	5 (36%)	4 (29%)	
**Distant metastasis**																				
No	185	151 (64%)	34 (14%)	0.166	195	100 (41%)	23 (9%)	0.432	65	37 (48%)	28 (36%)	0.329	63	34 (45%)	29 (39%)	0.434	9	8 (57%)	1 (7%)	0.052
Yes	50	34 (14%)	16 (8%)		51	95 (39%)	28 (11%)		12	5 (7%)	7 (9%)		12	5 (7%)	7 (9%)		5	2 (14%)	3 (22%)	
**Disease stage**																				
I+II	27	20 (8%)	7 (3%)	0.854	27	14 (6%)	13 (5%)	0.838	10	6 (8%)	4 (5%)	0.710	9	7 (9%)	2 (3%)	0.099	3	3 (21%)	0 (0%)	0.217
III+IV	218	165 (67%)	53 (22%)		219	109 (44%)	110 (45%)		67	36 (46%)	31 (41%)		66	32 (43%)	34 (45%)		11	7 (50%)	4 (29%)	
**Recurrence**																				
No	99	79 (32%)	20 (8%)	0.199	99	53 (22%)	46 (19%)	0.363	37	19 (25%)	18 (23%)	0.588	36	19 (25%)	17 (23%)	0.897	5	4 (29%)	1 (7%)	0.597
Yes	146	106 (43%)	40 (17%)		147	70 (28%)	77 (31%)		40	23 (30%)	17 (22%)		39	20 (27%)	19 (25%)		9	6 (43%)	3 (21%)	
**HPV status**																				
negative	237	179 (73%)	58 (24%)	0.973	238	119 (48%)	119 (48%)	1.000	72	39 (51%)	33 (42%)	0.800	70	36 (48%)	34 (45%)	0.711	12	8 (57%)	4 (29%)	0.334
positive	8	6 (2%)	2 (1%)		8	4 (2%)	4 (2%)		5	3 (4%)	2 (3%)		5	3 (4%)	2 (3%)		2	2 (14%)	0 (0%)	

According to the expression levels of the lactate transporters (MCT1 and MCT4) or the hypoxic biomarkers (HIF-1α and CAIX) in cancer cells, tumors were classified as hypoxic (Hx^+^) when over-expressed HIF-1α and/or CAIX regardless of the levels of MCT1 or MCT4 (142/245, 58%), and lactate-transporter tumors (LAc^+^) when overexpressed MCT1 and/or MCT4 regardless of the levels of HIF-1α or CAIX (51/75, 68%) (Table 4). The analysis of the hypoxic features revealed that the Hx^+^ phenotype was positively correlated with tumor size (*p* = 0.031). In addition, patients with Hx^+^-tumors had higher recurrence rate, including locoregional tumor control and distant metastases (HR: 1.387; 95% CI: 0.989–1.944, *p* = 0.052) than patients with tumors lacking over-expression of the hypoxia-markers (Hx^−^).

**Table 4:** Association between clinicopathological characteristics and the hypoxic or lactic phenotypes in tumors from patients with oropharyngeal SCCs

	hypoxic phenotype		*P*	lactic phenotype		*P*
	Hx^−^	Hx^+^		LAc^−^	LAc^+^	
**pT classification**						
T1 + T2	41	38	0.031	8	12	0.370
T3 + T4	62	104		16	39	
**pN clasiffication**						
N0	28	34	0.565	5	12	0.795
≥ N1	75	108		19	39	
**Histopathologic grade**						
well differentiated	48	63	0.766	10	21	0.968
poorly differentiated	55	78		14	30	
**Distant metastasis**						
No	87	108	0.107	21	42	0.571
Yes	16	34		3	9	
**Disease stage**						
I + II	10	17	0.577	4	5	0.394
III + IV	93	125		20	46	
**Recurrence**						
No	49	54	0.052	11	25	0.797
Yes	54	92		13	26	

The LAc^+^-tumor phenotype did not correlate with any clinicopathological features (Table 4). However, when LAc^+^ tumors were dichotomized into predominantly LAc^+^ tumors (33.33% of LAc^+^ tumors: LAc^+^Hx^-^) and tumors with mixed phenotype, Hx^+^ and LAc^+^ (LAc^+^Hx^+^ tumors (66.67% of LAc^+^ tumors: LAc^+^Hx^+^) depending on whether they lacked or harbored over-expression of HIF-1α/CAIX, respectively, the correlation analysis revealed a higher tendency to distant metastasis in LAc^+^Hx^+^ than in LAc^+^Hx^−^ -tumors (*p* = 0.034).

### Survival analysis

**Figure 6:**
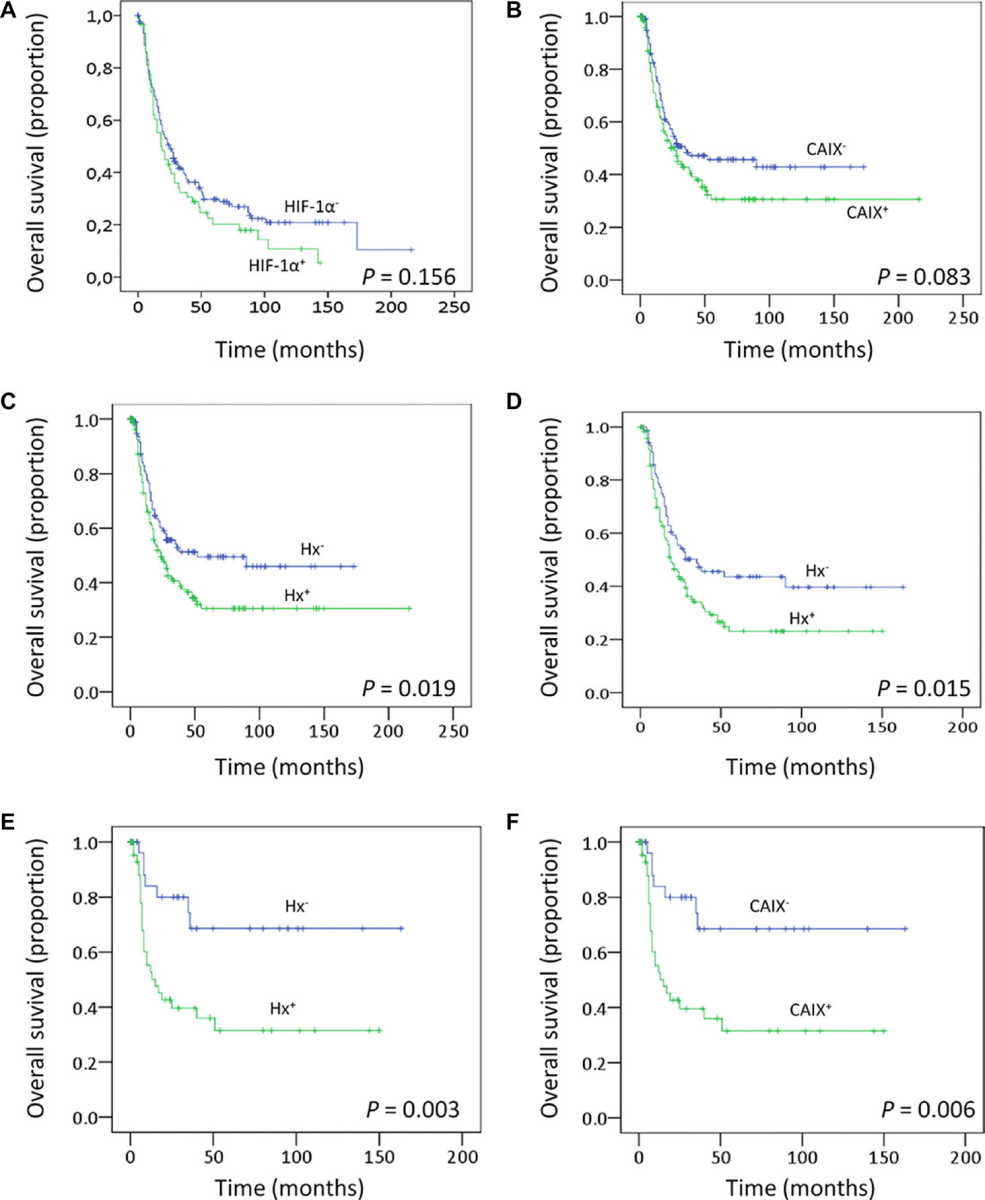
Clinical outcomes Kaplan–Meier estimates of overall survival among patients with oropharingeal SCCs that are classified according to the HIF-1α **(A)** or CAIX **(B)** expression levels or according to the Hx **(C)** phenotype. **(D–F)** Overall survival analysis including only those patients with oropharingeal SCCs disease stages III and IV (D) or patients that did not receive postoperative radiotherapy (E–F).

To evaluate the prognostic significance of clinicopathological variables and protein levels, Kaplan-Meier survival analysis was performed. As expected, large tumor size (*p* = 0.001) and disease recurrence (*p* < 0.001) were significantly associated with decreased overall survival (data not shown). Regarding protein expression levels, overall survival was lower in patients whose tumors expressed high levels of CAIX (*p* = 0.083) and also in patients with tumors over-expressing HIF-1α (*p* = 0.156), but the differences were not statistically significant (Figure 6A and 6B). In Hx^+^ tumors, a clear and significant difference in survival (*p* = 0.019, HR 1.43, 95% CI 1.05–1.94) was found, with high HIF-1α and/or CAIX expression predicting low overall survival (Figure 6C). A similar analysis for MCT1, MCT4 or LAc^+^ did not demonstrate a significant correlation to survival (data not shown), suggesting that HIF-1α/CAIX affects tumor aggressiveness beyond their co-expression with MCT1 or MCT4. A further analysis of only the high-stage tumors (stages 3 and 4) revealed that HIF-1α and/or CAIX immunostaining correlated with survival also in this material (*p* = 0.015), making HIF-1α and CAIX prognostic markers independent of the clinical staging (Figure 6D). When time to recurrence was introduced, in addition to age, tumor size, HPV status, and disease stage, in multivariate models to assess whether HIF-1α +/− CAIX over-expression has an independent prognostic meaning, statistical significance was lost (HR: 1.22, 95% CI: 0.88–1.7, *p* = 0.223). Thus, HIF-1α +/− CAIX over-expression, although is not an independent prognostic factor, they seem to somehow influence survival given that the trend toward low overall survival persisted.

The oxygenation status of the tumors is one of the known biomarkers for outcome of radiotherapy in head and neck SCCs [28]. Patients included in this study were all primarily treated by surgery but 67% received postoperative radiotherapy. We thus analyzed whether the clinical significance of the Hx^+^ phenotype is maintained in patients that exclusively received surgical treatment. The data showed that patients with Hx^+^-tumors or tumors expressing high levels of CAIX protein had higher recurrence rate (*p* = 0.006 and *p* = 0.071, respectively). In addition, high CAIX immunostaining (Figure 6F) and Hx^+^-phenotype (Figure 6E) correlated with low overall survival (*p* = 0.006 and *p* = 0.003, respectively). When tumor size, nodal metastasis, disease stage and recurrence were introduced in multivariate models to assess whether Hx^+^-phenotype has an independent prognostic meaning in surgically treated patients that did not received postoperative radiotherapy, statistical significance decreased to *p* = 0.220 although the trend toward low overall survival persisted (HR: 1.44; 95% CI: 0.796–2.69). As indicated above, HIF-1α +/− CAIX over-expression seems to somehow influence survival although it is not an independent prognostic factor.

## DISCUSSION

This study provides *in vivo* evidences supporting that HIF-1α, CAIX, miR-210 and ISCU are coordinately activated in oropharyngeal SCCs. In addition to CAIX, tumors expressing HIF-1α frequently express high levels of other pH-regulating enzymes, the lactate/H^+^ transporters MCT1 and MCT4 albeit co-expression in the same cancer cells only partially overlaps.

By using cell-based assays, our data revealed that hypoxic SCC cells over-express high levels of glycolytic genes [29] in a HIF-1α-dependent manner. We also confirmed that CAIX is one of the most highly over-expressed genes in hypoxic SCCs via a HIF-1α-dependent mechanism [30]. However, in contrast to other reports, over-expression of the lactate/H^+^ transporter, MCT4, was not detected in SCC cells exposed to hypoxia. In agreement with the *in vitro* data, the simultaneous analysis of CAIX and HIF-1α protein levels in SCC tumor tissues revealed that the two proteins are jointly co-expressed in cancer cells. HIF-1α and CAIX are also found co-expressed with MCT4 and MCT1 in some tumor nests but the overlapping was incomplete thus suggesting that the HIF-1α- and MCT1/MCT4-driven metabolic remodeling towards phenotypes consisting in hyperglycolytic cells and a lactate rich microenvironment, respectively, are independently regulated.

Importantly, our analysis disclosed that a set of miRNAs may be also involved in the metabolic reprogramming induced by HIF-1α in SCC-derived cells. We found that hypoxia induces, via a HIF-1α-dependent mechanism, expression of a small set of microRNAs, miR-155, miR-193b, miR-194, and miR-210 that have also been implicated in the metabolic reprograming of cancer cells [16, 31–33]. Among these, miR-210, the best known HIF-1α-related miRNA, was the most highly over-expressed miRNA. miR-210 is known to regulate the mitochondrial electron transport via down-regulation of genes such as ISCU, SDHD (succinate dehydrogenase subunit D) and NDUFA4 (subunit of the NADH-ubiquinone oxidoreductase complex) [34]. In the present report, we confirmed our previous findings that ISCU mRNA levels are inversely correlated with miR-210 levels in hypoxic SCC38 cells [26] and that HIF-1α downregulation using siRNAs abrogates ISCU hypoxic-regulation. Importantly, we also show that this pathway operates *in vivo*. miR-210 and ISCU RNA levels were found inversely correlated in oropharyngeal SCC tissues in association with HIF-1α and CAIX over-expression. To the best of our knowledge, this is the first *in vivo* evidence for coordinated HIF-1α, CAIX, miR-210, and ISCU expression in cancer. Expression of these proteins and miR-210 has been studied in a variety of tumor types including SCCs of different locations within the upper aero digestive track [35, 36]; however, evidences for the coordinated activation of the HIF-1α/miR-210/ISCU pathway has only been previously reported by our group in paragangliomas [26, 37], a rare benign neuroendocrine neoplasia. In these tumors, activation of that pathway was associated to a pseudohypoxic tumor phenotype defined by the presence of VHL gene mutations, and accumulation of the CAIX protein. AL Harris's group also described a coordinated upregulation of miR-210 and downregulation of ISCU in renal carcinomas [38] but, in contrast to the data in paragangliomas, activation of this pathway was not correlated with VHL mutations and, thus, the connection between HIF and miR-210/ISCU remains unknown in this type of cancer.

Although the functional consequences of the HIF-1α -CAIX/miR-210/ISCU activity has not been directly studied in the present report, the results of our analysis support the notion that HIF-1α-driven CAIX, and miR-210 alterations contribute to a more aggressive behavior of the tumors. Low overall survival and higher recurrence rate was found in patients with tumors that accumulate high levels of HIF-1α and/or CAIX (Hx^+^ tumors). Although in a limited number of patients, the correlations between high miR-210 levels and large tumor size and distant metastasis found in our study suggest that miR-210 expression contributes to tumor aggressiveness of Hx^+^ oropharyngeal tumors, a finding that is in agreement with previously reported data [36]. A number of studies have shown high tumor expression of HIF-1α as adverse prognostic features in head and neck SCCs [39], but this has not been a universal finding [40]. This is partially due to the fact that HIF-1α has different prognostic value in head and neck SCC of different geographical locations [41]. CAIX over-expression in cancer cells has also been extensively analyzed in patients with head and neck SCCs but its over-expression has been mostly associated with poor prognosis in patients that received radiotherapy and/or chemoradiotherapy [42–44]. In surgically treated patients, high expression of CAIX in stromal but not cancer cells has been found associated with adverse outcome in oral SCCs [45]. Thus, the novelty of our study lies in that the combined analysis of CAIX and HIF-1α to identify Hx^+^ tumors has allowed the definition of a group of surgically treated patients who have a poor prognosis and therefore may profit from treatment intensification. Furthermore, analysis of the functional significance of the LAc phenotype of tumors revealed that LAc^+^ tumors had a higher tendency to metastasize via hematologic system in the presence than in the absence of hypoxia. This is likely due to the tendency of cancer cells to move away from hostile hypoxic and acidic environment as previously demonstrated [46]. These data are in agreement with reports showing that lactic acidosis, in the absence of hypoxia, leads to a shift of energy utilization of breast cancer cells from glycolysis towards OXPHOS, contributing thus to favorable clinical outcomes [46].

Collectively, the data described here lead us to posit the existence of a functional link between HIF-1α, glycolysis, miR-210/ISCU axis, and extracellular acidification of hypoxic tumors. Besides the very well-known HIF-1α-driven hyperglycolytic phenotype that produces vast amounts of lactic acid, the activation of the miR-210/ISCU axis likely reduces the mitochondrial electron transport what contributes to the shift from oxidative phosphorylation to glycolytic metabolism that could lead, on one hand, to intracellular acidification of cancer cells and, on the other hand, to the generation of glycolytic intermediates that could be diverted into branched metabolic pathways allowing the generation of biosynthetic precursors required for tumor cell proliferation and invasion. To avoid damaging effect of prolonged intracellular acidosis, hypoxic cells activate the pH-regulating enzyme, CAIX, to return intracellular pH to slightly alkaline values while acidifies the extracellular media. Hypoxic cells may also over-express MCT4 and MCT1, likely via HIF-1α-independent mechanism, which favors the export of lactate/H^+^ thus contributing to the generation of the pH gradient. Whereas MCT4 is known to mainly functions as a lactate-export protein, MCT1 can equally export and import lactate. Another possible scenario is that MCT1, in glycolytic cancer cells that co-express MCT4, functions as an exporter of pyruvate leading to a reduction of the oxidative metabolism and an indirect stimulation of glycolysis, as previously suggested [47]. CAIX may also augment MCT1 transport activity by a non-catalytic interaction [48]. Thus, an overarching conclusion of our study is that the glycolytic rate in hypoxic SCC cells could be tuned by the direct transcriptional activation of glycolytic genes by HIF-1α, and the indirect reduction of oxidative phosphorylation mediated by the HIF-1α/miR-210/ISCU pathway or by the export of lactate via MCT4 and MCT1 and/or the export of pyruvate via MCT1. We propose that hypoxia, by itself, promotes the aggressive behavior of tumors and that the symbiosis between hypoxic-cell response and the pH regulating apparatus led by CAIX in cancer cells facilitates the metastatic cascade. Beyond contributing to a more precise knowledge of the metabolic programs employed by SCC cells we believe that our analysis has important implications for the development and the design of combination metabolic-targeting therapies against hypoxia and lactic acidosis which will be promising strategies for treatment of oropharyngeal SCC. Further comprehensive analysis of the metabolic consequences derived from the activation of the miR-210/ISCU pathway in SCC cancer cells is required to fully understand the metabolic reprogramming of cancer cells.

## MATERIALS AND METHODS

### Cell culture and treatments

The established SCC2 and SCC38 cell lines, kindly provided by Dr R Grenman (Department of Otolaryngology, University Central Hospital, Turku, Finland), were grown in DMEM supplemented with 10% fetal bovine serum, 100 units/ml penicillin, 200 μg/ml streptomycin, 2 mM-glutamine, 20 mM HEPES (pH 7.3), and 100 μM nonessential amino acids. Cell line authentication was performed by arrayCGH [22, 49]. Growth curve analysis was performed regularly, and no phenotype changes were observed through the duration of this study. To avoid cross-contamination and phenotype changes, these cell lines have not been maintained in long-term cultures.

For microarray and miRNA profiling, cells were treated with HIF-1α- or control-siRNAs. 24 h after transfection, cells were incubated under normoxic (21% O_2_) or hypoxic (1% O_2_) conditions for 16 h. Experimental quadruplicates were used for both, microarray and miRNA profiling. Hypoxic treatments were performed in a hypoxic incubator (HERAcell 150). siRNA treatments were performed with HIF-1α siRNA duplex oligonucleotides (Dharmacon) or siCONTROL Nontargeting pool (Dharmacon) as previously described [26]. Protein and mRNA analyses by western blot and RT-qPCR, respectively, revealed a substantial inhibition (80% when compared with control) of HIF-1α expression 48 hr after transfection.

### Tumor specimens

Tumor samples were obtained from 246 patients with oropharyngeal SCCs, diagnosed and treated between 1990 and 2009 in the Hospital Universitario Central de Asturias (Table 1). The median follow-up time of patients was 23 months. For RNA studies, fragments containing more than 70% tumor cells were obtained from the core of the tumor. Tumor specimens were snap-frozen at time of surgical resection and stored at −80°C in RNAlater (Ambion) until processed. The algorithm used to detect the presence of human papillomavirus (HPV) was previously described in detail [50]. Informed consent was obtained from all patients. The study was approved by the Ethical Committee of the Hospital Central of Asturias and the methods were carried out in accordance with the approved guidelines and the principles expressed in the Declaration of Helsinki.

### RNA extraction and microarrays

RNA was obtained from SCC38 and SCC2 cells, treated con siRNAs and subsequently incubated under hypoxic or normoxic conditions (4 individual samples for each experimental condition), with the *mir*Vana^™^ miRNA Isolation Kit (Ambion) according to the manufacturer's instructions. RNA quantity was assessed on a NanoDrop 1000 spectrophotometer (Thermo Scientific) and RNA quality was determined on a Bioanalyser 2100 (Agilent Technologies, Palo Alto, CA). Samples with an RNA integrity number (RIN) between 8 and 10 were used. RNA was hybridized to Affymetrix (SantaClara, CA) GeneChip Human Genome U133 Plus 2.0 Arrays. Raw signal intensities for each probe set in the CEL files were analyzed using version 6.5 of the Partek^®^ Genomics Suite^™^ (Partek Inc., MO, USA). The robust multiarray average (RMA) normalization method was used for RMA background correction, quantile normalization and medianpolish probe set summarization. Expression values were transformed to log2 before statistical analysis. Genes that were expressed differentially between normal and tumor samples were detected with one-way analysis of variance (ANOVA). The recovered *P*-values of the comparisons were then corrected using a step-up false discovery rate (FDR) value of 5%. The resulting list of significantly differentially expressed genes was filtered to include only genes that demonstrated 2-fold or greater up-regulation. To search for enrichment of specific biological pathways, the genes showing significantly differential expression between groups of samples were analyzed using the DAVID (Database for Annotation, Visualization, and Integrated Discovery) and the Kyoto Encyclopedia of Genes and Genomes (KEGG) pathways. For validation of the microarray data, CAIX and EGLN3 expression was evaluated by RT-qPCR in an independent set of SCC38 and SCC2 samples (data not shown).

### TaqMan low-density array (TLDA)

Megaplex profiling using human TaqMan Low Density miRNA Arrays (TLDA) (Applied Biosystems) was used to assay the expression of 369 miRNAs as described by the manufacturer. Briefly, 100 ng of total RNA, obtained from 4 individual samples of SCC38 and SCC2 cells treated with control or HIF-1α siRNAs and incubated under normoxic or hypoxic conditions, was used in the megaplex reverse transcription (RT) reaction containing about 450 miRNA-specific RT primers provided by the manufacturer. The RT product was mixed with 2× TaqMan Universal PCR Master Mix, No AmpErase UNG (Applied Biosystems) and loaded onto the TLDA containing the 48-plex PCR reaction mix. TLDAs were run on a 7900HT Thermocycler (Applied Biosystems) using Sequence Detection Systems (SDS) software version 2.3. A single TLDA was used per sample. Data analysis was performed using SDS RQ manager v1.2 (Applied Biosystems) which utilizes the delta-delta CT method [51]. The endogenous small nucleolar control RNA, RNU44, was used for normalization. miRNAs that were expressed differentially were detected with one-way analysis of variance (ANOVA) using the Partek Genmocis Suite. The recovered *P*-values of the comparisons were then corrected using a step-up false discovery rate value of 5%. The resulting list of significantly differentially expressed miRNAs was filtered to include only those that demonstrated 2-fold or greater up or down-regulation. For a hypoxia-inducible miRNA to be considered as a putative HIF-1α target, the following criteria were used: (a) HIF-siRNA should decrease the hypoxic induction of the miRNA in SCC38 cells and both, the normoxic and hypoxic miRNAs levels in SCC2 (b) SCC2 cells should express higher levels of the HIF-1α-miRNA target than SCC38 cells under normoxic conditions, and (c) HIF-1α-miRNA target should not necessarily increase by the hypoxic treatment in SCC2 cell because maximal stabilization and/or activation of HIF-1α has already been achieved in normoxia. Independent analysis of miR-210 levels in normal and tumor tissues was performed by RT-qPCR (Supplementary Figure 3) to establish a cutoff value.

### Immunohistochemistry and immunofluorescence

Formalin-fixed, paraffin-embedded tissues were cut into 3-μm sections and mounted on poly-L-lysine-coated slides (DakoCytomation). Antigen retrieval was performed by heating 20 minutes in a pressure cooker with Envision^™^ FLEX target retrieval solution pH 9. Tissue slides were incubated with the following primary antibodies: mouse IgG anti-HIF1α monoclonal antibody (Becton Dickinson Transduction Laboratories, Erembodegem, Belgium) at 1:50 dilution for 30 min, rabbit IgG anti-MCT4 (Santa Cruz Biotechnology, Inc.) at 1:100 dilution for 30 min, rabbit anti-MCT1 at 1:100 dilution for 30 min and rabbit anti-CAIX (Abcam) at 1:100 dilution for 30 min, rabbit polyclonal ISCU (Santa Cruz Biotechnology, Inc.) at 1:100 dilution for 30 min. For immunohistochemistry and for each antibody, all the slides were stained simultaneously in an automated horizontal slide-processing system (Dako Autostainer Plus). Negative controls with either an omission of the primary antibody or with a normal mouse IgG (Santa Cruz Biotechnology, Inc.) in the primary incubation were also included. The slides were digitalized on a Leica SCN400F scanner and analysed with the SlidePath Gateway LAN software. Images were analysed randomly by three of the authors without knowledge of clinicopathological data. Following published recommendations [41], staining was quantified using a score which combines the intensity (0, below the level of detection; 1, weak; and 2, moderate-strong) and the percentage of positive cells (0% to 100%). Scores were calculated by multiplying the staining intensity and the extension, as previously described [52]. Overall, HIF-1α immune-scores ranged from 0 to 170 (median value of 9 and mean value of 5); CAIX immune-scores ranged from 0 to 230 (median value of 19 and mean value of 34); MCT4 immune-scores ranged from 0 to 300 (median value of 150 and mean value of 139); and MCT1 immune-scores ranged from 0 to 160 (median value of 5 and mean value of 26). The median values were selected as the cutoff to define highly immunostained samples (above the cutoff) and weakly immunostained samples (below the cutoff). Two of the tissue samples were also analysed by double labeling immunofluorescence with anti-HIF-1α and anti-CAIX or with anti-HIF-1α and anti-MCT4. Anti-rabbit IgG Alexa Fluor 488 and anti-mouse IgG Alexa Fluor 555 were used as secondary antibodies at 1:500 dilutions for 1 h. Immunofluorescence stainings were analyzed on a Zeiss AxioObserver Z1 microscope (Carl Zeiss, Germany) with a Plan-Apochromat 40X/1.3 (NA = 1.3, working distance = 0.21mm) or Plan-Apochromat 63X/1.4 (NA = 1.4, working distance = 0.19mm) oil lens objective, a camera (AxioCam MRm; Carl Zeiss), and Apotome (ApoTome 2; Carl Zeiss).

### Statistical analysis

All statistical analyses were done using the SPSS statistical software version 19 (SPSS, Inc., Chicago, IL). The relationship between protein expression levels was analyzed using the Spearman's rho correlation. Correlations among pT classification, pN classification, histopathologic grade, distant metastasis, disease stage or recurrence, and protein expression were computed using the χ^2^ test. This test was also used to compare miR-210 and ISCU RNA levels and protein expression. Survival curves were generated using the Kaplan–Meier method. The median values of HIF-1α, CAIX, MCT1, MCT4 scores were used as the cutoff to classify patients. The statistical significance of the differences between groups was evaluated using the log-rank test. Multivariable analyses were performed with the Cox proportional hazards model. Statistically significant variables from the univariable analyses and variables likely to affect the outcome (T, N, disease stage and time to recurrence) were entered into the multivariable Cox proportional hazards regression model. *P* < 0.05 was considered statistically significant.

## SUPPLEMENTARY FIGURES


