# Supplementary material for: Clinically relevant HIF-1α-dependent metabolic reprogramming in oropharyngeal squamous cell carcinomas includes coordinated activation of CAIX and the miR-210/ISCU signaling axis, but not MCT1 and MCT4 upregulation
Source: Oncotarget. 2017 Jan 13;8(8):13730–46. doi: 10.18632/oncotarget.14629 (PMC5355133; doi:10.18632/oncotarget.14629)
Supplement: Supplementary file 1 [file oncotarget-08-13730-s001.pdf]

Clinically relevant HIF-1α-dependent metabolic reprogramming in oropharyngeal squamous cell carcinomas includes coordinated activation of CAIX and the miR-210/ISCU signaling axis, but not MCT1 and MCT4 upregulation

Supplementary Materials

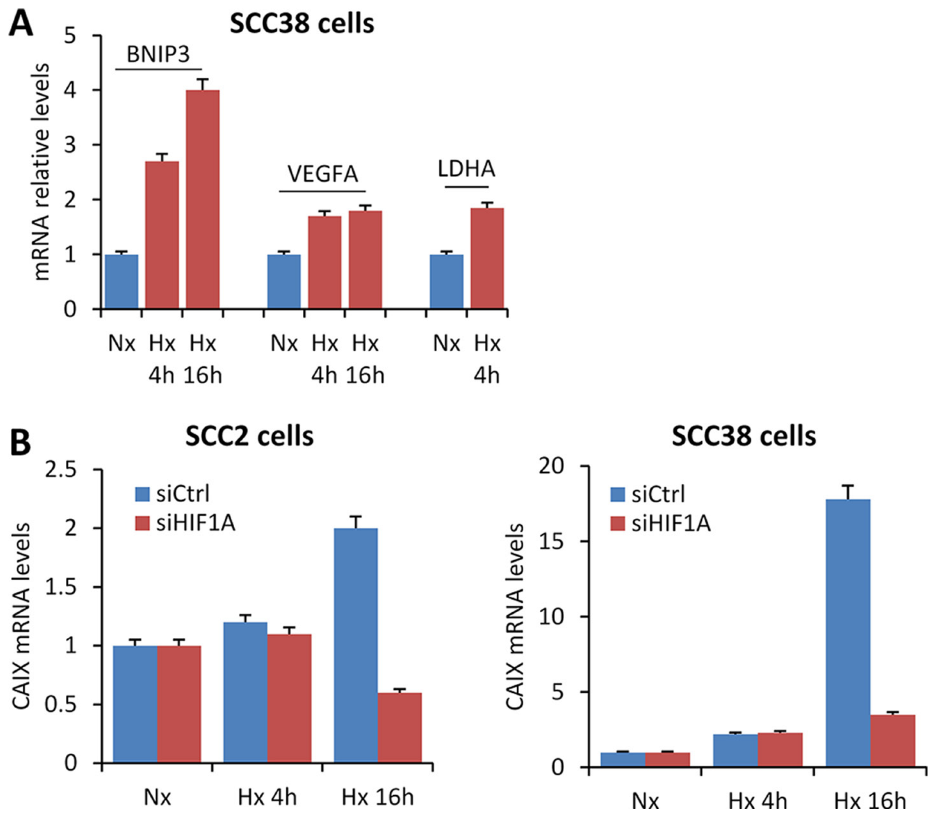

Supplementary Figure 1: Time-dependent expression of HIF-1α target genes in SCC-derived cell lines. (**A**) SCC cells were exposed to normoxia (Nx, 21% O_2) or hypoxia (Hx, 1% O_2) for the indicated periods of time before mRNA quantifications. (**B**) CAIX mRNA levels were analyzed in the indicated cells following treatment with HIF-1α (siHIF1A) or control (siCtrl) siRNAs. 24 hours after transfection, cells were incubated under normoxic (Nx) or hypoxic (Hx, 1% O_2) conditions for the indicated periods of time. Values are expressed as mean ratios ± SD.

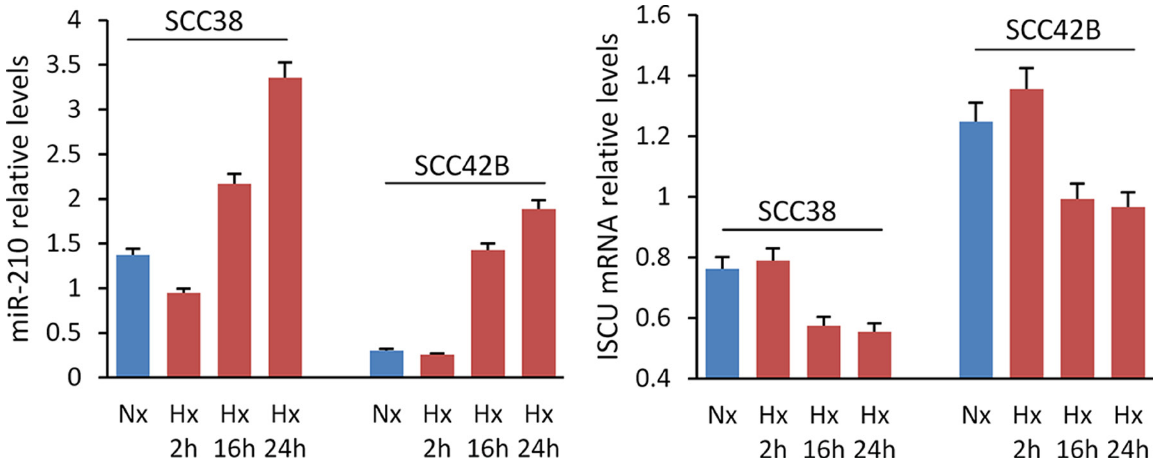

Supplementary Figure 2: Time-dependent expression of miR-210 and ISCU in SCC-derived cell lines. The indicated SCC cell lines were exposed to normoxia (Nx, 21% O_2) or hypoxia (Hx, 1% O_2) for the indicated periods of time before mRNA quantifications. miR-210 over-expression requires long-term periods of incubation to hypoxia (≥ 16 h), as expected for a transcriptional inductor of miR-210. Reduced levels of ISCU were detected at the same time points. Values are expressed as mean ratios ± SD.

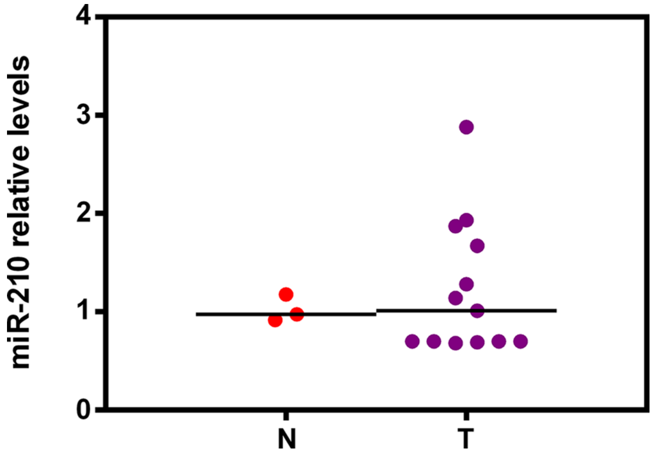

Supplementary Figure 3: Comparison of miR-210 levels in normal oropharyngeal mucosa and tumor tissue samples. miR-210 levels were quantified in 3 normal mucosa from non-cancerous patients and 14 tumor tissues.
